# Pattern of Progression of Oral Potentially Malignant Disorders From Real‐World Data: A 24‐Year Study

**DOI:** 10.1002/hed.70284

**Published:** 2026-04-29

**Authors:** Subin Surendran, Sumsum P. Sunny, Preethi M. Chathoth, William J. Magner, S. Lynn Sigurdson, Mihai Merzianu, Norbert Sule, Anurag K. Singh, Kimberly E. Wooten, Ryan P. McSpadden, Vishal Gupta, Amritha Suresh, Ashok Z. Samuel, Rohit Bhargava, Praveen N. Birur, Wesley L. Hicks, Moni Abraham Kuriakose

**Affiliations:** ^1^ Department of Head and Neck/Plastic and Reconstructive Surgery Roswell Park Comprehensive Cancer Center Buffalo New York USA; ^2^ Integrated Head and Neck Oncology Research Program, Mazumdar Shaw Medical Foundation, Narayana Health Bangalore India; ^3^ Department of Pathology Roswell Park Comprehensive Cancer Center Buffalo New York USA; ^4^ Department of Radiation Medicine Roswell Park Comprehensive Cancer Center Buffalo New York USA; ^5^ Beckman Institute for Advanced Science and Technology University of Illinois Urbana‐Champaign Urbana Illinois USA; ^6^ Karkinos Healthcare Mumbai Maharashtra India

**Keywords:** leukoplakia, malignant transformation, oral epithelial dysplasia, oral potentially malignant disorder, oral squamous cell carcinoma

## Abstract

**Background:**

Oral potentially malignant disorder (OPMD) prognostication is limited by uncertainty of which lesions will progress and the aggressiveness of lesion progression. We defined patterns and kinetics of progression in a large cohort of OPMD subjected to serial clinical and histologic examinations.

**Methods:**

This 24‐year study evaluated the pattern of disease progression in 668 patients with 1313 OPMDs.

**Results:**

The disease progression pattern was grouped as (a) non‐progressive (82.5%), (b) progressive, where initial low‐grade dysplasia advanced to high‐grade dysplasia in subsequent lesions (5.8%), and (c) transformed, where dysplastic lesions transformed into squamous cell carcinoma (11.7%). Progression showed aggressive (< 2 years) and indolent (> 2 years) patterns. An aggressive course occurred in 48.3% of progressive and 57.6% of transformed lesions. Progression was associated with high‐grade lesions, floor of mouth and palate subsites, and tobacco and alcohol use.

**Conclusions:**

The data from this study may be used to develop a risk‐stratified approach for OPMD management.

**Abbreviations**
CI: confidence interval
CIS: carcinoma in situ
EHR: electronic health records
FOM: floor of mouth
GBM: gingivo‐buccal mucosa
HGD: high‐grade dysplasia
HGL: high‐grade lesions
HR: hazard ratio
LGD: low‐grade dysplasia
LGL: low‐grade lesions
OED: oral epithelial dysplasia
OPMD: oral potentially malignant disorders
RMT: retromolar trigone
SCC: squamous cell carcinoma
SQL: Structured Query Language
WHO: World Health Organization

## 1 Introduction

Oral Potentially Malignant Disorders (OPMD) represent a spectrum of clinically and histologically defined conditions with varying risk of disease progression [1, 2]. A non‐dysplastic lesion may progress to invasive squamous cell carcinoma (SCC) passing through different grades of dysplasia. This linear progression model has been questioned as the lesions may regress, remain static, or progress from non‐dysplastic to various grades of dysplasia, and some lesions may transform to SCC [3]. The reported risk of OPMD malignant transformation varies widely in the literature, ranging from 0.13% to 34.0% [4, 5, 6, 7]. The kinetics of progression may vary from a few months to several years, making it a significant clinical challenge to introduce a reliable surveillance strategy for OPMD. Understanding the natural history and patterns of OPMD progression in a clinical setting where the patients may have undergone various interventions, such as habit cessation or surgical excision, is critical to developing real‐world evidence. This can guide clinicians to develop effective surveillance protocols, predict disease progression, and optimize timing for therapeutic intervention.

Warnakulasuriya and Kerr [8] have proposed a conceptual framework classifying oral cancer into four distinct groups based on the rate of disease progression as (1) aggressive, (2) less aggressive, (3) indolent, and (4) non‐progressing lesions. However, the relevance of such disease progression models in OPMD is yet to be established. Furthermore, the correlation of progression patterns with clinical and histopathological variables, such as lesion site, dysplasia grade, and evaluation of associated biomarkers, has not been systematically studied.

We hypothesized that OPMDs, based on their individual biological characteristics, may exhibit distinct patterns of disease progression over time with differing risks of malignant transformation. We also postulated that specific clinical and pathologic features may predict the disease trajectories. The objectives of this study were: (1) to identify disease progression patterns through long‐term follow‐up of a cohort of OPMD lesions from a single center, and (2) to identify key clinical and histopathologic factors associated with disease progression and malignant transformation.

## 2 Methodology

Patients with clinically suspicious OPMD presented to the Department of Head and Neck Surgery at Roswell Park Comprehensive Cancer Center (RPCCC), Buffalo, NY, USA, from January 2000 to June 2024, were included in the study. After obtaining Institutional Review Board approval (I‐281415), patients with OPMD were identified from the electronic health records (EHR) database by filtering for individuals with relevant pathology reports suggestive of OPMD using Structured Query Language (SQL) queries containing keywords of “dysplasia,” “erythroplakia,” “leukoplakia,” “hyperplasia,” “erythro‐leukoplakia,” “squamous cell carcinoma,” “microinvasive carcinoma,” “carcinoma in situ,” and terms indicating oral cavity subsites. Patients diagnosed with malignant tumors were identified within the Hospital Cancer Registry using SQL queries that employed specific ICD‐O‐3 codes for oral sites (C020‐C024, C028‐C029, C030‐C031, C039‐C041, C048‐C052, C058‐C062, C068).

Comprehensive patient data, including demographics and risk habit history were also compiled from both the EHR and the Hospital Cancer Registry. Furthermore, clinical notes and follow‐up dates were extracted from EHR documents and reviewed to ascertain the clinical and histologic disease status for each of the lesions. Subjects histopathologically diagnosed with OPMD and having at least two documented follow‐up visits were included in the study analysis. Those lesions without initial histopathologic confirmation were excluded from the study. Although the department does not have a fixed OPMD surveillance protocol, all patients with OPMD are kept on indefinite follow‐up with evaluation at 3‐to‐6‐month intervals. If there was clinical suspicion of lesion progression based on change in the lesion morphology from homogenous to non‐homogenous leukoplakia or erythroplakia, increase in lesion size or development of new lesions, the patients were advised to undergo incisional biopsy. All the biopsies were reviewed by one of two board‐certified pathologists specialized in head and neck oncology. The histopathological grading was reported as per the prevailing WHO grading criteria (2nd to 4th WHO editions, 1997, 2005 and 2017) [9, 10]. Accordingly, the tissue samples from biopsies were reported as hyperplasia with no dysplasia, mild, moderate, severe dysplasia and carcinoma in situ (CIS). For lesions that underwent more than one biopsy during the same visit, the higher grade was attributed to the grade of the lesion. The lesions were then regrouped histopathologically into low‐grade lesions (LGL: hyperplasia and mild dysplasia) and high‐grade lesions (HGL: moderate, severe dysplasia and CIS). The HGLs were recommended to undergo surgical excision.

The baseline clinical information obtained included age, gender, risk habits, lesion site and histological diagnosis (degree of dysplasia or carcinoma) at the baseline biopsy. Follow‐up outcomes tracked lesion duration and histopathology. These outcomes included clinical status of the lesion, any changes in dysplasia grade or malignant transformation of the same lesion, which were confirmed by biopsy. The lesions were categorized into four groups based on the pattern of disease progression: (1) De novo SCC, if evidence of invasive carcinoma was present at the initial biopsy; (2) Transformed, if the lesion transformed into invasive SCC on follow‐up, confirmed by histopathology; (3) Progressive OPMD, if dysplasia progressed from low to high grade during follow‐up, confirmed by histopathology; and (4) Non‐progressive OPMD, if the lesion remained unchanged or regressed on repeat histopathologic examination, or clinically regressed or remained static clinically on follow‐up. The progressive and transformed lesions were further classified into (a) aggressive, if the progression or transformation occurred during the first 2 years, and (b) indolent, if progression or transformation was seen more than 2 years after initial diagnosis.

### 2.1 Statistical Analysis

Descriptive statistics were used to define cohort characteristics. Chi‐square test was used to find the relationship between categorical variables. The Kaplan–Meier survival analysis and Cox regression were employed to identify predictors of transformation (Medcalc 23.2.1, MedCalc Software Ltd., Ostend, Belgium).

## 3 Results

### 3.1 Baseline Characteristics of Patient Cohorts

**FIGURE 1:**
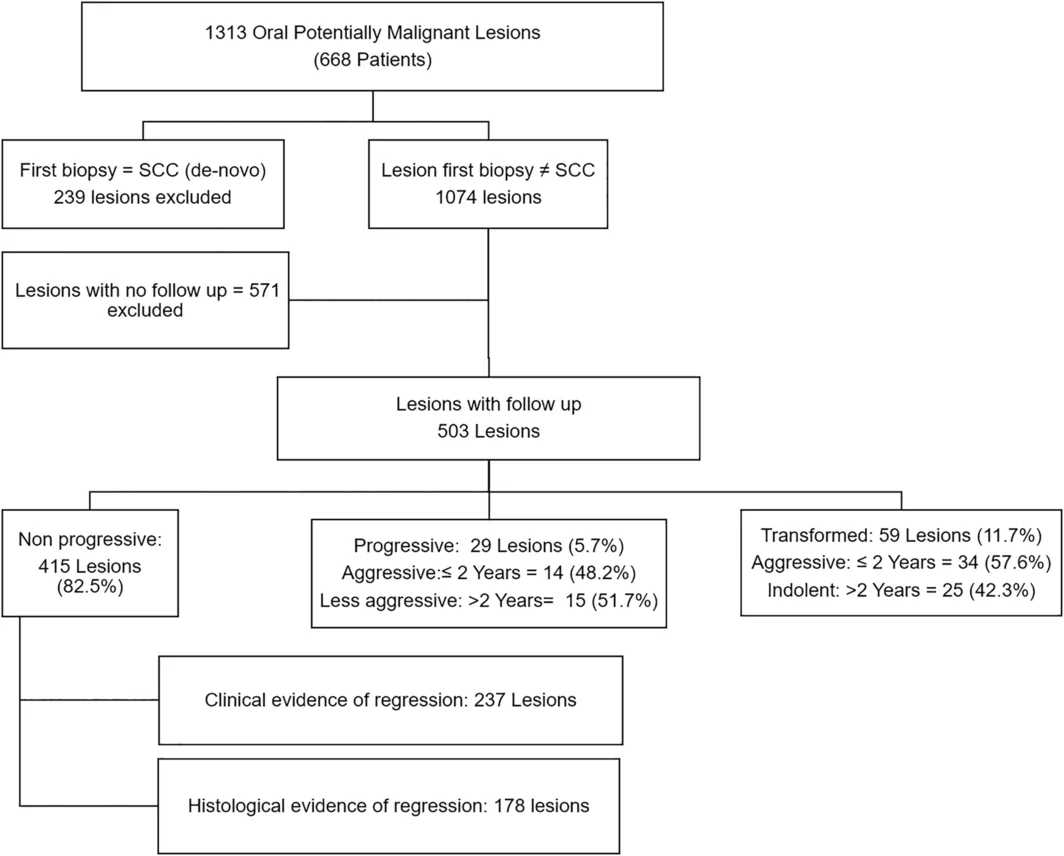
Study CONSORT diagram. From an initial 1313 oral lesions, 503 lesions from 255 patients were included for final analysis after excluding those diagnosed as squamous cell carcinoma (SCC) at baseline or lost to follow‐up. During the follow‐up period, 59 lesions (11.7%) underwent malignant transformation, 29 (5.8%) showed progressive changes, and 415 (82.5%) were non‐progressive.

The study cohort consisted of 668 patients with 1313 lesions initially suspected to be OPMD. Of these, 239 lesions were diagnosed as SCC at the baseline biopsy. These lesions were not included in the disease progression analyses. Five hundred and seventy‐one lesions without follow‐up information were also excluded from the analysis. The final follow‐up cohort comprised 503 lesions from 255 patients (Figure 1).

During follow‐up, 415 lesions were non‐progressive, of which 237 showed clinical regression without histological confirmation and 178 were histologically confirmed as non‐progressive, including 34 (19.1%) HGL and 144 (80.8%) LGL. Progressive changes were observed in 29 (5.8%) lesions, with 14 classified as aggressive (less than 2 years) and 15 as indolent (more than 2 years). Malignant transformation to SCC occurred in 59 lesions (11.7%), with 34 (57.6%) transforming within 2 years (aggressive), and 25 (42.3%) lesions after more than 2 years (indolent). During the follow‐up, in addition to clinical examinations, those lesions demonstrating clinical progression (increasing in size, change in morphology from homogeneous leukoplakia to erythro‐leukoplakia), and any new lesions were subjected to biopsy. A total of 863 serial biopsies from 503 lesions in 255 patients were carried out to monitor histopathologic status. Patient clinical and demographic characteristics at baseline are summarized in Table 1.

**TABLE 1:** Baseline clinical and demographic characteristics of oral lesions with clinical/biopsy follow‐up.

Characteristics	Number of patients	255 (lesions = 503)
Age (years)	Median (range)	60 (31–91)
Gender	Male	128 (50.2%)
	Female	127 (49.8%)
Habits	No habits	83 (32.6%)
	Drinking alcohol	37 (14.5%)
	Smoking	58 (22.8%)
	Smoking and other habits	77 (30.2%)
Oral subsites	Hard palate	19 (3.8%)
	RMT	17 (3.4%)
	Floor of mouth	52 (10.3%)
	Buccal mucosa	87 (17.3%)
	Gingiva	79 (15.7%)
	Tongue	249 (49.5%)
Diagnosis at baseline	No dysplasia	246 (48.7%)
	Mild	106 (21.1%)
	Moderate	59 (11.9%)
	Severe	52 (10.3%)
	Carcinoma in situ	40 (7.9%)

Abbreviation: RMT, retromolar trigone.

The median age of the OPMD patients included in the analysis (*n* = 255) was 60 years (range: 31–91 years), with a nearly equal gender distribution (Table 1). Tobacco use history was reported in 135 patients, while 83 had no known risk habits, and 37 reported alcohol consumption. The oral subsites involved were tongue (49.5%; *n* = 249), gingivo‐buccal mucosa (GBM) and retromolar trigone (36.4%; *n* = 183), floor of mouth (FOM) (10.3%; *n* = 52), and hard palate (3.8%; *n* = 19). The majority of the lesions were histopathologically classified as LGL, 70.0% (*n* = 352), comprising hyperplasia (*n* = 246) and mild dysplasia (*n* = 106). HGL accounted for 30.0% (*n* = 151), including moderate (*n* = 59), severe dysplasia (*n* = 52), and CIS (*n* = 40) (Figure S1).

#### 3.1.1 De Novo SCC

At the initial biopsy, 239 lesions from 198 patients were classified as invasive SCC and designated as de novo SCC. The median patient age of this group was 62 years (range: 26–91 years), with a higher prevalence observed among men (male‐to‐female ratio: 1.4:1; 115 males and 83 females). History of tobacco use was noted in 51.0% (*n* = 101) of the patients. The most frequently involved anatomical site was the tongue, accounting for 50.6% (*n* = 121) of the lesions, followed by the GBM, which comprised 25.1% (*n* = 60) of the lesions (Table 2).

**TABLE 2:** Demographics of de novo SCC.

Characteristics	Number of patients	198 (lesions = 239)
Age (years)	Median (range)	62 (26–91)
Gender	Male	115 (58.1%)
	Female	83 (41.9%)
Habits	No habits	53 (26.8%)
	Drinking alcohol	45 (22.2%)
	Smoking	31 (15.7%)
	Smoking and other habits	70 (35.3%)
Oral subsites	Hard palate	11 (4.6%)
	RMT	14 (5.9%)
	Floor of mouth	33 (13.8%)
	Buccal mucosa	23 (9.6%)
	Gingiva	37 (15.5%)
	Tongue	121 (50.6%)

Abbreviation: RMT, retromolar trigone.

### 3.2 Pattern of Disease Progression

Five hundred and three lesions from 255 patients were monitored for disease progression. The total duration of follow‐up ranged from 1 month to 252 months, with a median follow‐up period of 19 months.

#### 3.2.1 Non‐Progressive Lesions

Out of 503 lesions, 415 (82.5%) were non‐progressive, of which 237 (47.1%) regressed on clinical examination and in 178 (35.4%), regression was confirmed by repeat biopsy (Figure 1). Among lesions confirmed by biopsy, 60.1% (*n* = 107) remained as low‐grade, 19.1% (*n* = 34) remained as high‐grade, and regression (HGL regressed to LGL) was found in 20.8% (*n* = 37) of lesions (Table 3, Figure S2). The majority of lesions involved were oral tongue (48.4%) and GBM (35.1%). Among patients who had histological follow‐up with biopsy, the majority of GBM lesions (65.3%; 32/49) persisted as LGL, and most of the non‐progressing HGL lesions involved the oral tongue (61.8%; 21/34).

**TABLE 3:** Demographics of non‐progressive lesions.

Characteristics	Number of patients	228 (lesions = 415)
Age (years)	Median (range)	59 (31–91)
Gender	Male	111 (48.7%)
	Female	117 (51.3%)
Habits	No habits	72 (31.6%)
	Drinking alcohol	32 (14.0%)
	Smoking	48 (21.1%)
	Smoking and other habits	76 (33.3%)
Oral subsites	Hard palate	15 (3.6%)
	RMT	14 (3.4%)
	Floor of mouth	39 (9.4%)
	Buccal mucosa	73 (17.6%)
	Gingiva	73 (17.6%)
	Tongue	201 (48.4%)
Diagnosis at baseline	No dysplasia	203 (48.9%)
	Mild	82 (19.8%)
	Moderate	55 (13.3%)
	Severe	44 (10.6%)
	Carcinoma in situ	31 (7.5%)

Abbreviation: RMT, retromolar trigone.

#### 3.2.2 Progressive Lesions

Progressive lesions, defined as an increase in dysplasia grade at follow‐up, were identified in 5.8% (29/503) of lesions confirmed by histopathologic examination. The demographics and clinical features of progressive lesions are summarized in Table 4. An attempt was made to identify any association of clinical and pathological characteristics with the pattern of disease progression. Comparing progressive and non‐progressive lesions, most of the progressive lesions were associated with tongue (18/29, 62.1%) and lesions that did not have high‐risk habits (14/29, 48.3%) (Figure S3).

**TABLE 4:** Demographics of progressive lesions.

Characteristics	Number of patients	26 (lesions = 29)
Age (years)	Median (range)	62 (34–90)
Gender	Male	10 (38.5%)
	Female	16 (61.5%)
Habits	No habits	12 (46.2%)
	Drinking alcohol	2 (7.7%)
	Smoking	4 (15.4%)
	Smoking and other habits	8 (30.8%)
Oral subsites	RMT	1 (3.4%)
	Floor of mouth	2 (6.9%)
	Buccal mucosa	6 (20.7%)
	Gingiva	2 (6.9%)
	Tongue	18 (62.1%)
Diagnosis at baseline	No dysplasia	17 (58.6%)
	Mild	12 (41.4%)

Abbreviation: RMT, retromolar trigone.

#### 3.2.3 Malignant Transformation

**FIGURE 2:**
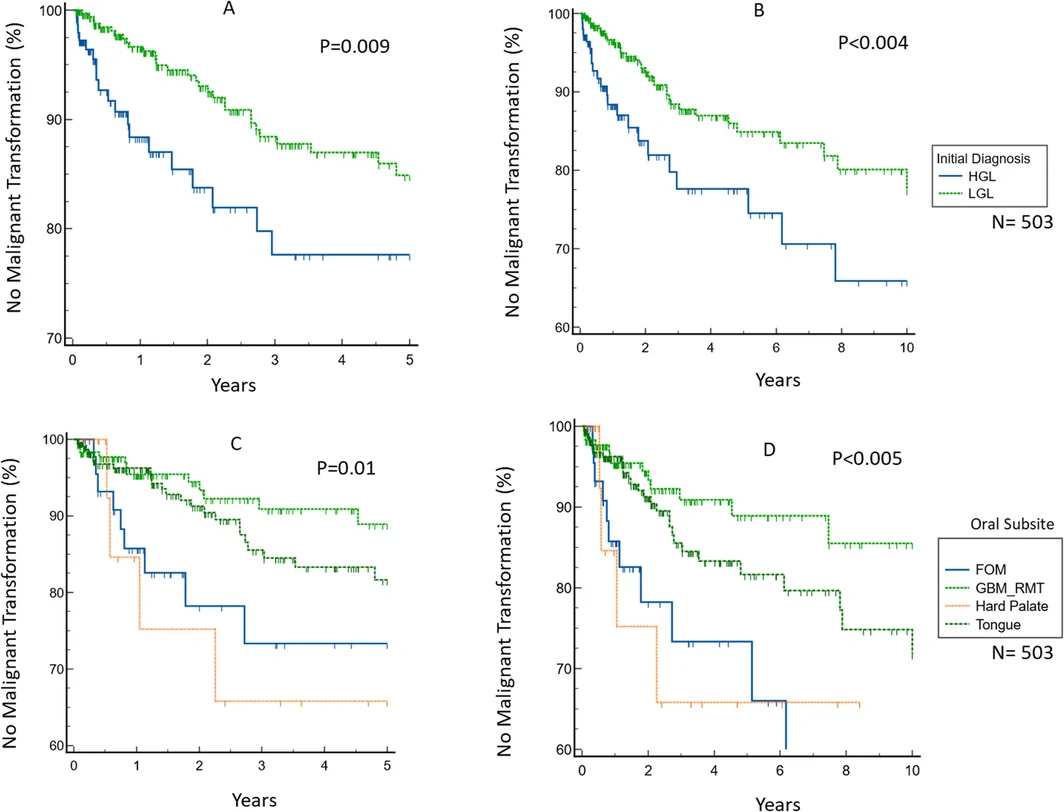
Kaplan–Meier analysis of OPMD malignant transformation. Kaplan–Meier analysis demonstrated the impact of baseline histological grade and anatomical site. High‐grade lesions showed significantly higher malignant transformation rate over a 5 years (A) and 10 years (B) follow‐up. Site‐specific analysis showed that lesions located on the floor of mouth and hard palate were associated with a significantly greater risk of malignant transformation compared with lesions on the tongue and gingivobuccal complex/retromolar trigone (GBM/RMT) (C, D).

Malignant transformation was observed in 11.7% (59/503) of the lesions. Malignant transformation was more common in tongue lesions (30/59) and was seen in patients with high‐risk tobacco or alcohol habits (40/59). Gender and age were not associated with disease transformation (Table 5, Figures S4 and S5). The higher rate of malignant transformation of HGL (14.6%) was statistically significant compared to LGL (10.5%; *p* = 0.004), (Figures 2A,B and S6).

**TABLE 5:** Demographics of transformed lesions.

Characteristics	Number of patients	54 (lesions = 59)
Age (years)	Median (range)	61 (36–88)
Gender	Male	26 (48.1%)
	Female	28 (51.9%)
Habits	No habits	14 (25.9%)
	Drinking alcohol	5 (9.3%)
	Smoking	10 (18.5%)
	Smoking and other habits	25 (46.3%)
Oral subsites	RMT	2 (3.4%)
	Hard palate	4 (6.8%)
	Floor of mouth	11 (18.6%)
	Buccal mucosa	8 (13.6%)
	Gingiva	4 (6.8%)
	Tongue	30 (50.9%)
Histology	No dysplasia	25 (42.4%)
	Mild dysplasia	12 (20.3%)
	Moderate dysplasia	5 (8.5%)
	Severe dysplasia	8 (13.6%)
	Carcinoma in situ	9 (15.3%)

Abbreviation: RMT, retromolar trigone.

### 3.3 Kinetics of Disease Progression and Malignant Transformation

**FIGURE 3:**
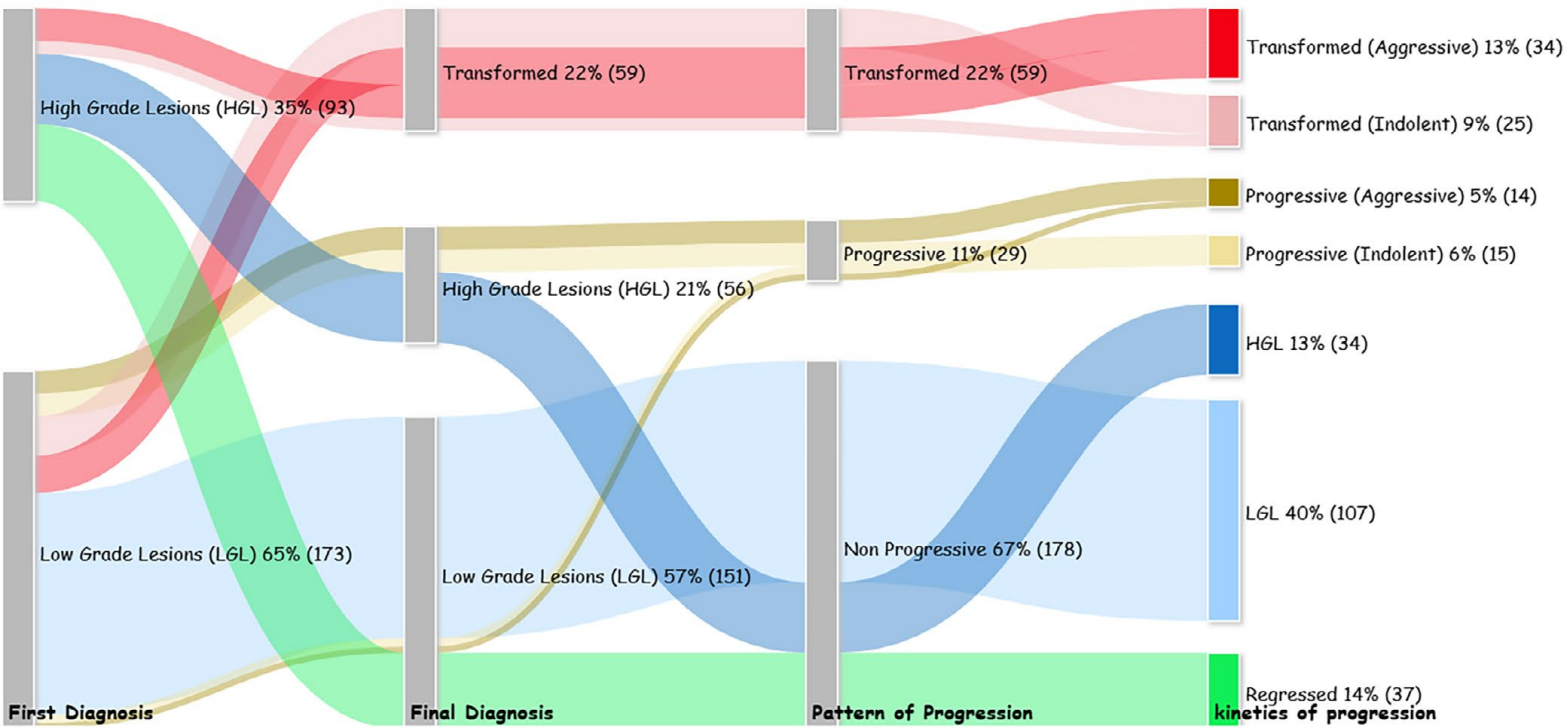
Pattern of histological progression in oral potentially malignant disorders with histological follow up (*n* = 266). Sankey diagram illustrates the longitudinal pattern of OPMD from initial biopsy diagnosis through final outcomes, progression patterns and kinetics. At baseline, 65.0% of lesions were low‐grade lesions (LGL) and 35.0% were high‐grade lesions (HGL). On follow‐up, 57.0% remained LGL/HGL, 21.0% progressed to HGL, while 22.0% (59 lesions) transformed to carcinoma. Overall, 67.0% of lesions were non‐progressive, 14.0% regressed, 11.0% showed progressive changes, and 22.0% underwent malignant transformation. This included both aggressive (< 2 years) (13.0% transformed, 5.0% progressive) and less aggressive (> 2 years) (9.0% transformed, 6.0% progressive).

Among the progressive lesions, 48.3% (*n* = 14/29) demonstrated aggressive behavior (progression within 2 years), while 51.7% (*n* = 15/29) showed an indolent pattern (progression after 2 years) (Figure 3). In the lesions that underwent malignant transformation, 57.6% (34/59) progressed to carcinoma in less than 2 years. The aggressive transformation phenotype dominated among patients with a smoking history (23/34) as well as those with lesions of the FOM (8/11) and hard palate (3/4), Figure S7.

Kaplan–Meier survival analyses revealed that lesions initially diagnosed as HGL demonstrated a significantly higher rate of malignant transformation compared to LGL over 5 years (Hazard Ratio = 2.40; 95.0% CI: 1.24–4.7, *p* = 0.009) and 10 years (Hazard Ratio = 2.45; 95.0% CI: 1.32–4.57, *p* = 0.004) periods (Figure 2A,B). A similar trend was observed when cases were further stratified by grades of dysplasia (Figure S6). The FOM lesions demonstrated a significantly higher malignant transformation rate compared to GBM (10 years: Hazard Ratio: 3.5; 95.0% CI: 1.35–9.21; *p* = 0.005; 5 Years: Hazard Ratio: 3.06; 95.0% CI: 1.10–8.50; *p* = 0.01) (Figure 2C,D). In contrast, demographic and clinical variables including age, gender and risk habit history did not show any significant association with the aggressiveness of malignant transformation (Figure S5). When kinetics of lesion progression were correlated with dysplasia grade, aggressive behavior of lesions was more associated with HGL (aggressive = 16/34 and indolent = 6/25) than LGL (*p* = 0.07).

## 4 Discussion

The natural history of OPMD may be perturbed by different interventions such as risk habit cessation and surgical excision. Understanding the pattern of disease progression from real‐world data is essential to develop an effective surveillance strategy and interventions for OPMD. The present study was carried out in a large cohort of OPMD managed in a tertiary care cancer hospital and attempted to identify the pattern of disease progression where the patients have undergone standard of care treatment and followed up for a long duration.

**FIGURE 4:**
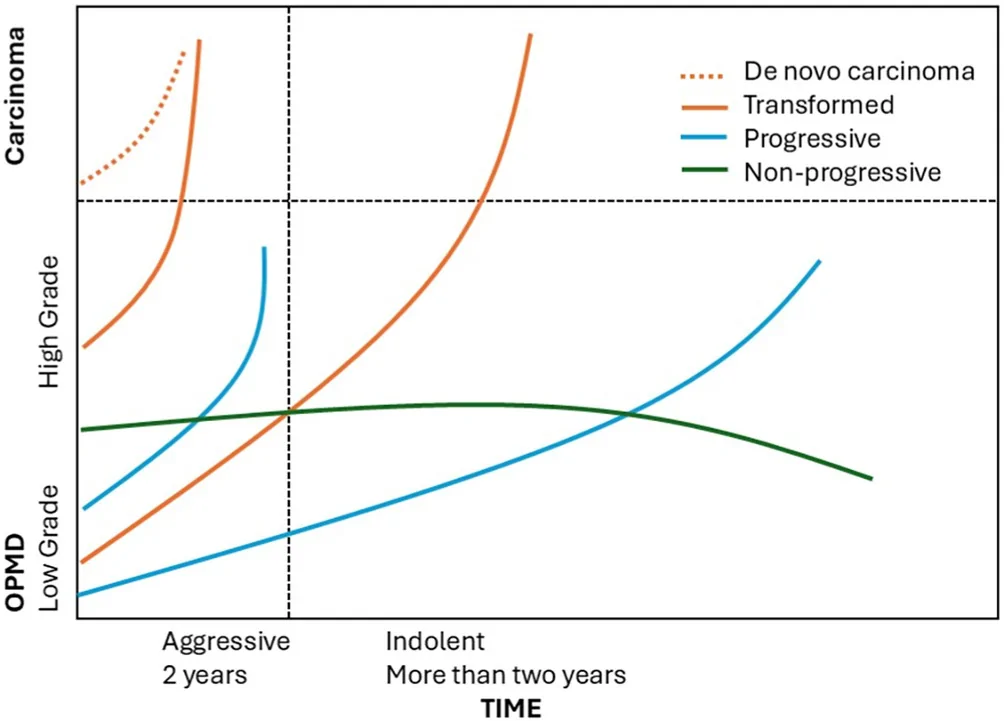
Patterns of progression of OPMD. Lesions presenting as OPMD upon biopsy may harbor invasive carcinoma (de novo carcinoma) or undergo malignant transformation (transformed) with an aggressive or indolent course, or lesions may progress to a higher grade of dysplasia (progressive) following an aggressive or indolent course or may remain static or regress with time (non‐progressive). Adapted from Warnakulasurya and Kerr [8].

Currently, there exists no established follow‐up protocol for OPMDs [9]. The recommended follow‐up frequency ranges from 3 to 6 months and the duration is not well defined. In the current study of 1313 histologically confirmed OPMD reviewed over a period of 24 years and the lesions confirmed by histology, we were able to decipher four patterns of disease progression: *de novo* carcinoma, non‐progressive, progressive, and transformed. The progressive and transformed lesions followed either an aggressive (less than 2 years) or indolent (more than 2 years) clinical course (Figure 4).

Many early oral cancers present as a flat superficial lesion. A high index of suspicion is essential for the early diagnosis of these lesions [11]. The high prevalence of carcinoma at the initial diagnosis (18.2%, 239/1313) seen in this cohort is expected as the study was carried out in a tertiary cancer care setting. This underscores the importance of careful clinical examination and biopsy of all oral lesions presenting as OPMD. This approach is not only necessary to detect cancer at an early stage, but also for baseline characterization of the OPMD lesions and to plan risk‐stratified treatment and surveillance.

Among the lesions with follow‐up, malignant transformation was observed in 11.7%, which is consistent with previous studies. Two systematic reviews reported a wide range of malignant transformation (0.13% to 34.0%) [4, 5] with a pooled malignant transformation rate of 6.6%. The variability of malignant transformation in these reports was correlated with the sample size, follow‐up time, risk factors, clinical practice settings and diagnostic criteria used for OPMD.

Identifying lesion features associated with risk of disease progression and malignant transformation potential, as well as anticipated time for disease progression are essential to select lesions appropriate for a wait and see (surveillance) option or an immediate surgical intervention. In our study, among those lesions that progressed (from low‐grade to higher grade of dysplasia), about half showed aggressive behavior (within 2 years), and the other half were indolent (after 2 years). This suggests that an intense surveillance protocol needs to be adopted for the high‐risk lesions during the first 2 years and periodically thereafter. The low‐risk lesions also need to be followed up long‐term; however, with a less intense regimen. The two‐years period to define aggressive lesions in this study was selected empirically to conform with the oral cancer surveillance protocol [12, 13]. Those lesions that remain asymptomatic during follow‐up, any suspicious change in morphology biopsy should be performed.

Determinants of malignant transformation have been extensively studied in the past. They include site, size, duration, grade of dysplasia and the presence of risk factors [14]. The present study concurred with these findings. The high‐risk patients identified in this study included those with HGL (moderate, severe dysplasia and CIS) and ongoing risk habits, a point supported by prior studies [14, 15, 16, 17]. WHO periodically updates the criteria for defining and grading oral epithelial dysplasia. We have used the prevailing WHO criteria and the grading system and did not make efforts to reclassify the lesions based on the current grading system. Although our inclusion of “carcinoma in situ” reflects the prevailing grading system during the study period, it is to be noted that the current WHO classification has removed “carcinoma in situ” cohort and recommended to incorporate these lesions as severe dysplasia. FOM and tongue have long been considered high‐risk sites [18]. However, in our study, while the tongue exhibited a high disease progression rate, this site was not associated with a statistically significant increase in the rate of malignant transformation to SCC. Whether the lower rate of malignant transformation of this subsite is a result of surgical intervention of HGL per our protocol cannot be ruled out. Lesions of the FOM and hard palate appear to be a strong indicator of aggressive behavior and malignant transformation (Figure S7).

The presence of dysplasia is generally considered to confer malignant transformation potential to OPMD. Dost et al. [19] examined the malignant transformation of oral epithelial dysplasia (OED) and found that 7.1% of their study population underwent progression or malignant transformation with an annual malignant transformation rate of 1%. Their study questioned the predictive value of OED grading for malignant transformation, suggesting that the severity of dysplasia did not correlate with malignant transformation. It is to be noted that in the present study, non‐dysplastic and low‐grade dysplasia did undergo disease progression and malignant transformation (10.0% and 11.0%, respectively). This fact was highlighted in a US population study [20], which underscores the limitation of dysplasia as a marker for malignant transformation. We and others have shown that OSCC occurs in the setting of low‐grade dysplasia or no dysplasia in about one third of patients [21]. In the current study, as well as observations from previous reports [18], it can be stated that, although grade of dysplasia does not determine the overall malignant transformation of OPMDs, high‐grade lesions are more likely to follow aggressive clinical behavior. Recognition of this finding is especially significant for the management of an individual patient presenting with OPMD. The finding also highlights the necessity to develop other prognostic biomarkers to predict disease progression. In this respect, based on multiple variables, a dynamic nomogram was developed by Cai et al. to predict the malignant transformation of OPMDs and to implement interventions [22].

Warnakulasuriya et al. emphasized that even clinically normal‐appearing mucosa in patients harboring potentially malignant lesion(s) may have dysplasia, emphasizing the importance of careful examination of the entire oral cavity. However, malignant transformation, if it were to happen, is likely to occur in the index lesion rather than in the normal mucosa. Therefore, attention to the lesion is especially important during surveillance [23]. Currently, there is no consensus on criteria for intervention in OPMD with dysplasia [24]; but in most practices, as well as in our center, HGL are treated by surgical excision when feasible.

Based on our findings, it is clear that the majority of OPMD lesions do not progress to cancer (82.5%); in fact, a subset of the lesions showed clinical regression (14%). This finding suggests that factors such as lifestyle, local treatment, or even spontaneous resolution may influence the course of OPMDs. The active surveillance program to detect early cancer adopted in our center likely plays a role in the high number of biopsies identified as LGL. These factors need to be considered to develop a risk‐based surveillance protocol for OPMD. The recommended clinical surveillance protocol for OPMD is summarized in Table 6. It is to be noted that the setting of this study was in a comprehensive cancer center and not in a community clinic. As a tertiary care center, most of the patients evaluated in this study would have had initial consultations in the community and likely received counseling on tobacco and alcohol cessation. In addition, the high‐risk lesion patients in the cancer center would have undergone excision. These interventions may have affected disease progression; nevertheless, our study provides real‐world clinical evidence addressing the presence of varied OPMD progression patterns. It also highlights the importance of careful clinical and histological confirmation at baseline and during follow‐up visits to identify aggressive lesions.

**TABLE 6:** Surveillance protocol of OPMD based on risk profile.

Risk type	Features	Surveillance protocol
Low risk OPMD	Site: Alveolus and buccal mucosa Size: < 2 cm Clinical Diagnosis: Homogenous leukoplakia Grade of dysplasia: no dysplasia or low grade	Every 6 months till disease regression Biopsy of any new lesions or evidence of clinical progression
High risk OPMD	Site: Floor of mouth, palate and tongue Size: > 2 cm Clinical Diagnosis: Non‐homogenous leukoplakia and erythroplakia Grade of dysplasia: moderate and severe grade	Every 3 months review till the disease become low‐risk OPMD and then continue surveillance every 6 months till disease regression Biopsy of any new lesions, or evidence of clinical progression

## 5 Conclusion

OPMD exhibits diverse patterns of clinical course including regression, non‐progression/stable disease, progression or malignant transformation. Disease progression and transformation were associated with HGL, continuation of risk habits, and lesions located in the FOM and palate. These features were also associated with an aggressive clinical course with progression in less than 2 years. Lesions with no dysplasia and mild dysplasia also have long‐term malignant transformation potential. Clinical management of OPMD requires intense surveillance in the first 2 years and long‐term disease monitoring along with the development of novel prognostic biomarkers to predict the risk of malignant transformation.

## Funding

This work was supported by the Roswell Park Comprehensive Cancer Center and National Cancer Institute (NCI) grant P30CA016056.

## Conflicts of Interest

The authors declare no conflicts of interest.

## Supporting information


**Figure S1:** Clinicopathological characteristics and outcomes of oral potentially malignant lesions with follow up (255 Patients, 503 Lesions).
**Figure S2:** The distribution of non‐progressive lesions according to gender, habit and subsite.
**Figure S3:** Distribution of progressive lesions by gender, risk habits, and oral subsite.
**Figure S4:** Distribution of transformed OPMD by gender, risk habit, and oral subsite.
**Figure S5:** Malignant transformation–free survival stratified by demographic factors and risk habit history. Kaplan–Meier curves depict over follow‐up time (years) in the cohort (*N* = 503). (A, B) Transformation‐free survival by age group (< 50 vs. ≥ 50 years) shown over 5 years (A) and 10 years (B) follow‐up. (C, D) Transformation‐free survival by gender (female vs. male) is shown over 5 years (C) and 10 years (D) follow‐up. Transformation‐free survival by risk habit history (categories as indicated in the legend) shown over 5 years (E) and 10 years (F) follow‐up. *p*‐values shown are from log‐rank tests comparing curves within each panel. No statistically significant association was found between the aggressiveness of malignant transformation and demographic variables such as age, gender, and habit history.
**Figure S6:** Malignant transformation–free survival stratified by baseline (initial) diagnosis. Kaplan–Meier curves show over follow‐up time (years), grouped by initial diagnosis: carcinoma in situ (CIS; red), mild dysplasia (blue), moderate dysplasia (black), no dysplasia (green), and severe dysplasia (magenta). (A, B) Analysis in the full cohort (*N* = 503) displayed over 5 years (A) and 10 years (B) follow‐up. (C, D) Analysis in a sub‐cohort (*N* = 266, lesions having histology follow‐up) displayed over 5 years (C) and 10 years (D). *p*‐values shown are from the log‐rank test. Hazard ratios (HRs) with 95% confidence intervals (CI) shown below each panel are from Cox proportional hazards comparisons between (significant) diagnostic groups. These analyses are exploratory and underpowered due to insufficient sample size.
**Figure S7:** The Sankey diagram traces the longitudinal course of OPMD lesions by oral site, risk habits and gender. While majority (67%) of OPMD remained non‐progressive, the remaining 33% underwent histological progression or malignant transformation following distinct aggressive (< 2 years) or indolent (> 2 years) patterns. The tongue was the most common site (58.0%), followed by gingivobuccal mucosa/retromolar trigone (27.0%), floor of mouth (FOM) (10.0%), and hard palate (5.0%). Smoking alone (21.0%) or smoking with other habits (36.0%) was associated with malignant transformation. The cohort showed a nearly equal gender distribution, with a slight female predominance (52.0%).

## Data Availability

The data that support the findings of this study are available from the corresponding author upon reasonable request.
