# Supplementary material for: Pattern of Progression of Oral Potentially Malignant Disorders From Real‐World Data: A 24‐Year Study
Source: Head Neck. 2026 Apr 29;48(10):2800–9. doi: 10.1002/hed.70284 (PMC13595463; doi:10.1002/hed.70284)
Supplement: Supplementary file 1 [file HED-48-2800-s001.docx]

**Supplementary document:**

**Pattern of progression of oral potentially malignant disorders from real-world data: a twenty-four-year study**

Subin Surendran PhD^1^***, Sumsum P Sunny MDS PhD^2^***, Preethi M Chathoth BS ^1^, William J. Magner PhD^1^, S. Lynn Sigurdson PhD^1^, Mihai Merzianu MD^3^, Norbert Sule MD^3^, Anurag K. Singh MD^4^, Kimberly E. Wooten MD^1^, Ryan P. McSpadden MD^1^, Vishal Gupta MD^1^, Amritha Suresh PhD^2^, Ashok Z Samuel PhD^5^, Rohit Bhargava PhD^5^, Praveen N Birur MDS^2^, Wesley L. Hicks Jr MD^1^, Moni Abraham Kuriakose MD^1,2,6^**^#^**

**The two authors contributed equally to this study*

#Corresponding Author

**Affiliations**

^1^Department of Head and Neck/Plastic and Reconstructive Surgery, Roswell Park

Comprehensive Cancer Center, Buffalo, New York, USA, 14263

^2^Integrated Head and Neck Oncology Research Program, Mazumdar Shaw Medical

Foundation, Narayana Health, Bangalore, India 560099

^3^ Department of Pathology, Roswell Park Comprehensive Cancer Center, Buffalo, New

York, USA, 14263.

^4^ Department of Radiation Medicine, Roswell Park Comprehensive Cancer Center,

Buffalo, New York, USA, 14263.

^5^ Beckman Institute for Advanced Science and Technology, University of Illinois

Urbana- Champaign, Urbana, IL, USA 61801

^6^Karkinos Healthcare, Mumbai, Maharashtra, India, 400086

**^#^**Moni Abraham Kuriakose, MD
Department of Head and Neck/Plastic and Reconstructive Surgery,

Roswell Park Comprehensive Cancer Center, Buffalo, New York, 14263

moni.kuriakose@roswellpark.org

**Funding:** This work was supported by the Roswell Park Comprehensive Cancer Center and National Cancer Institute (NCI) grant P30CA016056.

**Running Title:** Patterns and kinetics of OPMD progression

**Keywords:** Oral Squamous Cell Carcinoma; Oral Potentially Malignant Disorder; Oral Epithelial Dysplasia; Malignant Transformation; Leukoplakia

**Supplementary Figures:**

**Supplementary Figure 1. Clinicopathological characteristics and outcomes of oral potentially malignant lesions with follow up (255 Patients, 503 Lesions).**

Age distribution shows non-progressive lesions peaked between the fifth to seventh decades. Gender demonstrates near equal distribution, with females (127, 49.8%) and males (128, 50.2%). Risk habit history depicts the distribution of patient risk habits: 22.4% reported smoking alone, 47% reported multiple risk habits, and 30.2% had no habits. Lesion site distribution, with the tongue being the most common site (49.5%), followed by gingivobuccal mucosa/retromolar trigone (36.4%), floor of mouth (FOM) (10.3%), and hard palate (3.8%). Histological diagnosis at baseline, where nearly half of the cases showed no dysplasia (48.7%), while mild (21.1%), moderate (11.9%), severe dysplasia (10.3%), and carcinoma in situ (CIS) (7.9%). Mean follow up duration across categories, with progressive lesions having the longest average follow up (3.9 years), compared to non-progressive and transformed lesions (2.7 years each).

**Supplementary Figure 2. The distribution of non-progressive lesions according to gender, habit and subsite.**

(A) Lesions were almost equally distributed between males and females. (B) Among LGL, the most common exposure was smoking plus other habits followed by smoking alone; HGL showed similar associations, with alcohol plus others, smoking plus others and no habit (C) LGL/HGL most commonly involves the tongue followed by gingivobuccal mucosa/retromolar trigone.

**Supplementary Figure 3. Distribution of progressive lesions by gender, risk habits, and oral subsite.**

(A)Gender, (B) Habit associations and (C) Oral subsite distribution shows no significant association between aggressive and indolent categories.

**Supplementary Figure 4. Distribution of transformed OPMD by gender, risk habit, and oral subsite.**

(A) Gender distribution shows aggressive lesions and less aggressive lesions similarly distributed by gender. (B) Habit associations reveal that aggressive lesions were predominantly associated with smoking exposure (C). Oral subsite distribution indicates that aggressive lesions were most frequent on the tongue.

**Supplementary Figure 5. Malignant transformation–free survival stratified by demographic factors and risk habit history.** Kaplan–Meier curves depict over follow-up time (years) in the cohort (N = 503). (A,B) Transformation-free survival by age group (<50 vs ≥50 years) shown over 5-year (A) and 10-year (B) follow-up. (C, D) Transformation-free survival by gender (female vs male) is shown over 5-year (C) and 10-year (D) follow-up. Transformation-free survival by risk habit history (categories as indicated in the legend) shown over 5-year (E) and 10-year (F) follow-up. P-values shown are from log-rank tests comparing curves within each panel. No statistically significant association was found between the aggressiveness of malignant transformation and demographic variables such as age, gender, and habit history.

**Supplementary Figure 6: Malignant transformation–free survival stratified by baseline (initial) diagnosis.** Kaplan–Meier curves show over follow-up time (years), grouped by initial diagnosis: carcinoma in situ (CIS; red), mild dysplasia (blue), moderate dysplasia (black), no dysplasia (green), and severe dysplasia (magenta). (A, B) Analysis in the full cohort (N = 503) displayed over 5-year (A) and 10-year (B) follow-up. (C, D) Analysis in a sub-cohort (N = 266, lesions having histology follow-up) displayed over 5-year (C) and 10-year (D). P-values shown are from the log-rank test. Hazard ratios (HRs) with 95% confidence intervals (CI) shown below each panel are from Cox proportional hazards comparisons between (significant) diagnostic groups. These analyses are exploratory and underpowered due to insufficient sample size.

**Supplementary Figure 7. The Sankey diagram traces the longitudinal course of OPMD lesions by oral site, risk habits and gender.** While majority (67%) of OPMD remained non-progressive, the remaining 33% underwent histological progression or malignant transformation following distinct aggressive (<2 years) or indolent (>2 years) patterns. The tongue was the most common site (58.0%), followed by gingivobuccal mucosa/retromolar trigone (27.0%), floor of mouth(FOM) (10.0%), and hard palate (5.0%). Smoking alone (21.0%) or smoking with other habits (36.0%) was associated with malignant transformation. The cohort showed a nearly equal gender distribution, with a slight female predominance (52.0%)
